# The p23 of Citrus Tristeza Virus Interacts with Host FKBP-Type Peptidyl-Prolylcis-Trans Isomerase 17-2 and Is Involved in the Intracellular Movement of the Viral Coat Protein

**DOI:** 10.3390/cells10040934

**Published:** 2021-04-17

**Authors:** Zuokun Yang, Yongle Zhang, Guoping Wang, Shaohua Wen, Yanxiang Wang, Liu Li, Feng Xiao, Ni Hong

**Affiliations:** 1 Key Lab of Plant Pathology of Hubei Province, College of Plant Science and Technology, Huazhong Agricultural University, Wuhan 430070, China; 13297974203@163.com (Z.Y.); zhangyongle@webmail.hzau.edu.cn (Y.Z.); gpwang@mail.hzau.edu.cn (G.W.); wenshaohua1985@163.com (S.W.); wangyanxiang@webmail.hzau.edu.cn (Y.W.); 15032210591@163.com (L.L.); fengxiao@webmail.hzau.edu.cn (F.X.); 2 Key Laboratory of Horticultural Crop (Fruit Trees) Biology and Germplasm Creation of the Ministry of Agriculture, Wuhan 430070, China; 3 National Biopesticide Engineering Research Centre, Hubei Biopesticide Engineering Research Centre, Hubei Academy of Agricultural Sciences, Wuhan 430064, China

**Keywords:** citrus tristeza virus, p23, coat protein, FK506-binding protein, protein-protein interaction

## Abstract

*Citrus tristeza virus* is a member of the genus *Closterovirus* in the family *Closteroviridae*. The p23 of citrus tristeza virus (CTV) is a multifunctional protein and RNA silencing suppressor. In this study, we identified a p23 interacting partner, FK506-binding protein (FKBP) 17-2, from *Citrus aurantifolia* (CaFKBP17-2), a susceptible host, and *Nicotiana benthamiana* (NbFKBP17-2), an experimental host for CTV. The interaction of p23 with CaFKBP17-2 and NbFKBP17-2 were individually confirmed by yeast two-hybrid (Y2H) and bimolecular fluorescence complementation (BiFC) assays. Subcellular localization tests showed that the viral p23 translocated FKBP17-2 from chloroplasts to the plasmodesmata of epidermal cell*s* of *N. benthamiana* leaves. The knocked-down expression level of NbFKBP17-2 mRNA resulted in a decreased CTV titer in *N. benthamiana* plants. Further, BiFC and Y2H assays showed that NbFKBP17-2 also interacted with the coat protein (CP) of CTV, and the complexes of CP/NbFKBP17-2 rapidly moved in the cytoplasm. Moreover, p23 guided the CP/NbFKBP17-2 complexes to move along the cell wall. To the best of our knowledge, this is the first report of viral proteins interacting with FKBP17-2 encoded by plants. Our results provide insights for further revealing the mechanism of the CTV CP protein movement.

## 1. Introduction

*Citrus tristeza virus* (CTV), a member of the genus *Closterovirus* in the family *Closteroviridae* [1], is phloem-restricted and naturally infects some citrus species and their relatives [2]. The virus has a single-stranded, positive-sense genomic ~19.3 kb RNA (gRNA), containing 12 open reading frames (ORFs) potentially encoding for at least 19 proteins [1,3,4]. ORFs 1a and 1b are expressed directly from the genomic RNA and encode replication-related proteins, and ORFs 2-11 are expressed via 3′-coterminal subgenomic RNAs [2,5]. Four proteins, including p65 (heat shock 70 protein homolog, HSP70h), p61, p27 (minor coat protein, CPm), and p25 (coat protein, CP), are involved in virion assembly and movement [6,7,8,9,10,11,12]. Proteins p25, p20, and p23 have RNA silencing suppressor (RSS) activities [13]. At present, there is no homolog of p23 found in other viruses in the family *Closteroviridae*. The CTV p23 contains a Zn finger domain, is an RNA binding protein, and plays a role in the asymmetrical accumulation of positive and negative RNA strands during CTV gRNA replication [14,15,16]. The p23 accumulates in the nucleolus, Cajal bodies, and plasmodesmata (PD) in agroinfiltrated leaves of *Nicotiana benthamiana* [17]. The p23 is also a pathogenic factor that elicits CTV-like symptoms of ectopically transgenic citrus plants [13,18,19]. Both RSS activity and pathogenicity of p23 appear to be related to its nucleolar localization. In addition, p23 might be involved in the long-distance movement of CTV by targeting intercellular filaments [17]. The CTV CP localizes to the cytoplasm and nuclei, and can inhibit cell-to-cell silencing by preventing the spread of silencing signals, and is also involved in interactions with host plants [13,20,21].

The successful infection of a plant virus greatly depends on the molecular interactions between the virus and its hosts [22]. The development of infectious clones of the CTV genotype T36 has greatly improved the study on the interactions of CTV with its natural host *Citrus* spp. and experimental herbaceous host *N. benthamiana* [23,24,25,26]. In recent years, extensive studies have been performed on the interactions between CTV and its host and the functions of CTV proteins [24,27,28,29]. However, the interaction between CTV proteins and host factors has hardly been investigated. Identifying host factors interacting with CTV proteins is helpful for understanding of the molecular mechanisms involved in virus pathogenesis. Recently, one study identified a CTV p23 interacting protein cytosolic glyceraldehyde 3-phosphate dehydrogenase (GAPDH) from an expression library of *N. benthamiana* by yeast two-hybrid (Y2H) and found that virus-induced silencing of GAPDH mRNA resulted in a decrease of CTV titer [30].

The FK506 binding proteins (FKBPs) are the receptors of FK506 (Tacrolimus), a macrolide antibiotic with immunosuppressive properties [31,32]. Plants have a large number of FKBPs, which function in a diversity of cellular processes, including stress response, development, transcription regulation, and chloroplast function in plants [33]. FKBPs are characterized as having at least one FK506-binding domain (FKBd), the receptor site for proline, and the active site for the peptidyl-prolyl cis-trans isomerase (PPIase) catalysis [33]. Proteomic analysis revealed a large amount of FKBPs associated with thylakoid membranes and lumens in *Arabidopsis thaliana* [34]. *A. thaliana* alone encodes for 23 FKBPs. Among AtFKBPs, 2 target the secretory pathway, 4 target the cytosol, 11 target the chloroplast, and 6 target the nucleus [35]. FKBP13 and FKBP16 are located in chloroplasts and regulate photosynthetic membrane assembly [25]. Previously, we constructed a cDNA library of *C. aurantifolia* and a CTV p23 interacting host protein was identified in a yeast two-hybrid assay [36]. Sequence analysis revealed that the p23-interacting protein was a homolog of FKBP 17-2 from *A. thaliana* and *Oryza sativa* (GenBank ID: NP_564048.1 and XP_015631550.1) [32,35]. The function of FKBP17-2 is still unclear. In this study, we cloned the cDNAs of FKBP17-2 gene from *C. aurantifolia*, a natural host of CTV [37], and *N. benthamiana*, an experimental host of a CTV-T36 infectious clone [26]. The interaction of FKBP17-2 and CTV p23 was confirmed by two independent methods. Further analysis demonstrated that FKBP17-2 also interacts with the CP of CTV and is involved in the movement of CTV CP in *N. benthamiana* leaf cells. Our results provided the first evidence that a plant FKBP homolog has the capacity to interact with two proteins of CTV and helps transportation of viral coat protein.

## 2. Materials and Methods

### 2.1. Virus Source and Plant Material

The CTV isolate N21, which is kept in *C. aurantifolia* plants in an insect-proof greenhouse [38], was used for the gene cloning of the virus. The infectious cDNA clone of the CTV isolate FS577 (GenBank ID: KC517488), which was driven by the cauliflower mosaic virus (CaMV) 35S promoter in a binary vector pCAMBIA1380, was kindly donated by Dr. Yan Zhou (National Citrus Engineering Research Center, Citrus Research Institute, Southwest University, Chongqing, China). Plants of *N. benthamiana* (wild-type) were grown in a chamber in 60% relative humidity with a 16 h daylight period at 25 °C during the day and an 8 h dark period at 20 °C during the night.

### 2.2. Gene Cloning

Primers used for the amplification of genes coding for proteins p23 and CP of CTV were designed based on multiple alignments of the CTV sequences available in GenBank. Primers used for the amplification of FKBP17-2 coding genes were designed based on cDNA sequences of CaFKBP17-2 from *C. aurantifolia* and NbFKBP17-2 from *N. benthamiana*, respectively. Total RNA was extracted using TRIzol reagent (Invitrogen, Carlsbad, CA, USA) according to the manufacturer’s instructions. The isolated RNA was used for first-strand cDNA synthesis via reverse transcription with the Maloney murine leukemia virus reverse transcriptase (M-MLV, Promega, Madison, WI, USA) and p(N)6 random primer (TaKaRa, Dalian, China) at 37 °C for 2 h. The PCR products were purified and ligated into the pMD18-T vector (TaKaRa, Dalian, China). Clones were identified via PCR and at least three positive clones of each product were sequenced at Sangon Biotech (Shanghai) Co., Ltd., Shanghai, China. One clone of each gene with a consensus sequence was selected for further study. The primers are listed in Table S1. GenBank accession numbers of genes analyzed in this study are as follows: *CaFKBP17-2* (MW600634), *NbFKBP17-2* (MW600635), CTV *p23* (MW600636), and CTV *CP* (EF063109.1).

### 2.3. DUALMembrane Yeast Two-Hybrid Assay

The DUALmembrane yeast two-hybrid (mYTH) system was used for testing protein-protein interactions following the manufacturer’s protocols (Dualsystems Biotech AG, Switzerland). The cDNAs of CaFKBP17-2 and NbFKBP17-2 were individually cloned into the library vector pPR3-N by fusing to the C-terminal half of Ubiquitin (Cub), and p23 was fused to the N-terminal half of Ubiquitin (NubG) of the bait vector pDHB1. The recombined pPR3-N and pDHB1 plasmids were co-transformed into *Saccharomyces cerevisiae* strain NMY51. The transformed yeast cells were grown on plates containing selection medium lacking tryptophan and leucine (SD-Trp,-Leu) (Clontech, Mountain View, CA, USA) at 30 °C for 2–4 days. The surviving clones were transferred to quadruple dropout medium lacking tryptophan, leucine, histidine and adenine (SD-Trp,-Leu,-His,-Ade) (Clontech, Mountain View, CA, USA) in the presence of 50 mM 3-amino-1,2,4-triazole (3-AT) (Sigma-Aldrich, St. Louis MO, USA). The self-interaction of CTV p20 was used as a positive control.

### 2.4. BiFC and Subcellular Localization Assays

Gateway Technology (Invitrogen, Carlsbad, CA, USA) was used for the construction of BiFC vectors. To facilitate subsequent Gateway cloning, primer sequences were flanked with *att*B recombination sites at their 5′ ends (Table S1). PCR products containing *att*B sites were gel-purified and recombined into the pDONR/Zeo vector using the Gateway™ BP Clonase™ Enzyme Mix (Invitrogen, Carlsbad, CA, USA). The genes in the pDONR/Zeo vectors were recombined into the modified pEarlygate201-YN or pEarlygate202-YC vectors [39] using the Gateway™ LR Clonase™ II Enzyme mix (Invitrogen, Carlsbad, CA, USA).

For subcellular localization assays, the genes coding for enhanced yellow fluorescent protein (eYFP) and enhanced cyan fluorescent protein (eCFP) were ligated into vector pCNF3 at the *Kpn*I and *Sma*I sites [40] to generate plant expression constructs 2× 35S-MCS-eYFP (pCNY) and 2× 35S-MCS-eCFP (pCNC), respectively. Genes were amplified from the corresponding clones by using primers with an *Xba*I or *BamH*I digestion site (Table S1), digested with enzymes *Xba*I and *BamH*I and inserted into vector pCNY or pCNC. The mCherry-HDEL vector was used as an endoplasmic reticulum (ER) marker [41]. The plant expression constructs were individually transformed into cells of *Agrobacterium tumefaciens* strain GV3101 by using a heat shock method. The GV3101 cells harboring the expression constructs were resuspended in the infiltration buffer (10 mM MgCl_2_, 10 mM 2-(4-morpholino) ethanesulfonic acid, and 150 mM acetosyringone) and infiltrated into *N. benthamiana* leaves, as previously described [42]. Callose deposition at plasmodesmata of infiltrated *N. benthamiana* samples were stained with a water solution of 0.05% (wt/vol) aniline blue (Coolaber, Beijing, China) at 30 min before image taking as previously described [43].

Fluorescence signals in infiltrated leaf sections were captured under a confocal laser scanning microscopy (CLSM; Leica Microsystems, TCS-SP8, Germany). eCFP, eYFP, and mCherry were excited at 458 nm, 514 nm, and 561 nm, respectively. Emission signals were captured at 470–500 nm, 530–585 nm, and 590–630 nm, respectively. Chlorophyll autofluorescence signals were excited at 561 nm and captured at 680–720 nm. When eYFP and mCherry were co-expressed, eYFP signals were excited at 514 nm and captured at 530–560 nm. Aniline blue signals were excited at 405 nm and captured at 448–525 nm.

Videos were taken using the time series program in TCS-SP8 CLSM. Collected images were analyzed with the Leica Application Suite for Advanced Fluorescence (LAS AF Lite, version 3.3) software.

### 2.5. Virus-Induced Gene Silencing (VIGS) and CTV Inoculation

VIGS vectors pTRV1 and pTRV2 were used for the RNA silencing analyses [44]. A fragment of *NbFKBP17-2* cDNA (position 80–296 nt of the gene) was amplified from *N. benthamiana* and then cloned into pTRV2. The resulting plasmid pTRV2-FKBP17-2 was electroporated into *A. tumefaciens* cells and co-infiltrated with an *A. tumefaciens* culture harboring pTRV1 (final optical density of 0.7 at OD600) into leaves of 4-week old *N. benthamiana* plants. At 7 dpi, leaves of *NbFKBP17-2* silenced plants and control plants were agroinfiltrated with CTV infectious clone.

### 2.6. Real-Time RT-PCR

Total RNA was extracted from *N. benthamiana* samples using TRIzol reagent (Invitrogen, Carlsbad, CA, USA). The cDNA was prepared using 5× All-In-One RT MasterMix (with AccuRT Genomic DNA Removal Kit) (Abm, VAN, Canada).

The relative expression levels of CTV genome and NbFKBP17-2 mRNA were tested using RT-qPCR. RT-qPCR was performed in a CFX96™ Real-time system (Bio-Rad, Hercules, CA, USA) using the i*Taq*^TM^ Universal SYBR^®^ Green Supermix (Bio-Rad, Hercules, CA, USA). CTV-specific primers amplifying the intergenic region between the RNA-dependent RNA polymerase and p33 ORFs were used for RT-qPCR assays of the CTV genome quantity [45]. The *N. benthamiana* actin gene (accession number AY179605) was used as an internal control. Values for relative FKBP17-2 expression was calculated by the 2^-^^ΔΔ^^C^_T_ method [46] and were normalized to the transcriptional value of an actin gene [38,47]. Results were obtained from three independent assays. The primers for RT-qPCR are listed in Table S1.

### 2.7. Sequence Analyses

Multiple sequence alignments were performed using the Muscle program in MEGA 7.0.26 software [48]. Conserved domains were predicted using NCBI CD-Search (https://www.ncbi.nlm.nih.gov/Structure/cdd/wrpsb.cgi, accessed on 12 August 2015).

## 3. Results

### 3.1. CTV p23 Interacts with FKBP17-2 In Vivo and In Vitro

**Figure 1:**
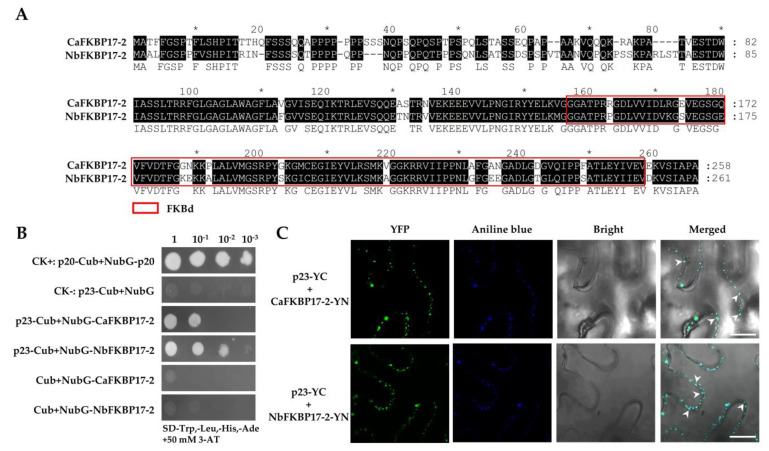
Interactions between citrus tristeza virus (CTV) p23 and FKBP17-2. (**A**) Alignment of deduced amino acid sequences of CaFKBP17-2 and NbFKBP17-2. Shaded letters indicate the conserved amino acid residues. The FK506 binding domain (FKBd) was indicated by a red box. (**B**) Split-ubiquitin yeast two-hybrid assays. p23 was fused to the C-terminal half of ubiquitin (Cub). CaFKBP17-2 and NbFKBP17-2 were respectively fused to the N-terminal half of ubiquitin (NubG). The yeast cell of co-transformed with p20-Cub and NubG-p20 was used as a positive control, and the yeast cell with p23-Cub and NubG was used as a negative control, respectively. A series of dilutions (1, 10^−1^, 10^−2^, and 10^−3^) were shown at the top of the figure. (**C**) Bimolecular-fluorescence complementation assays. The p23 fused to YFP C-terminal (p23-YC) was separately co-expressed with CaFKBP17-2 and NbFKBP17-2 fused to YFP N-terminal (CaFKBP17-2-YN and NbFKBP17-2-YN) by agroinfiltration on *N. benthamiana* leaves. Aniline blue stained callose at plasmodesmata (PD) was used as a marker. Arrow heads pointed to PD. Confocal images were taken at 48 hpi. Bars, 20 μm.

The full-length cDNAs coding for NbFKBP17-2 and CaFKBP17-2 were cloned from *N. benthamiana* and *C. aurantifolia*, respectively. NbFKBP17-2 and CaFKBP17-2 shared 82% amino acid identity and each contained a FKBd (Figure 1A). Yeast split-ubiquitin assays showed that the yeast cells transformed with paired vectors p20-Cub and NubG-p20 (a positive control), p23-Cub and NubG-CaFKBP17-2, and p23-Cub and NubG-NbFKBP17-2 were able to grow on selective media (SD-Trp,-Leu,-His,-Ade) complemented with 50 mM 3-AT, whilst two yeast transformants individually carrying p23-Cub+NubG and Cub+NubG-FKBP17-2 were unable to grow (Figure 1B), indicating that the p23 can interact with both CaFKBP17-2 and NbFKBP17-2.

The interactions of p23 with CaFKBP17-2 and NbFKBP17-2 were further investigated using the BiFC assay in *N. benthamiana* leaves. In this assay, p23 was fused to the C-terminal fragment of YFP (p23-YC), and CaFKBP17-2 and NbFKBP17-2 were respectively fused to the N-terminal fragment of YFP (CaFKBP17-2-YN and NbFKBP17-2-YN). The co-expression of p23-YC and CaFKBP17-2-YN, and p23-YC and NbFKBP17-2-YN via agroinfiltration resulted in extensive YFP fluorescence signals in the cytoplasm of agroinfiltrated cells at 48 h post infiltration (hpi). The YFP fluorescence signals of p23 interacting with CaFKBP17-2 and NbFKBP17-2 emerged as discontinuous spots along the cell membranes and granular structures in the cytoplasm, further staining of the infiltration leaf sections showed that these spots colocalized with the blue signals of aniline blue stained callose at PD (Figure 1C), but did not move in the cytoplasm under time-lapse microscopy observation (Figure S1). The results further confirmed that the interactions between the CTV protein p23 and FKBP17-2 proteins from *C. aurantifolia* and *N. benthamiana*, and their interaction signals mainly happened at PD. There was no interaction signal observed for the control combinations of p23-YC with unfused YN and FKBP17-2-YN with unfused YC (Figure S2).

### 3.2. Subcellular Localization of p23 and NbFKBP17-2 in N. benthamiana Leaf Cells

**Figure 2:**
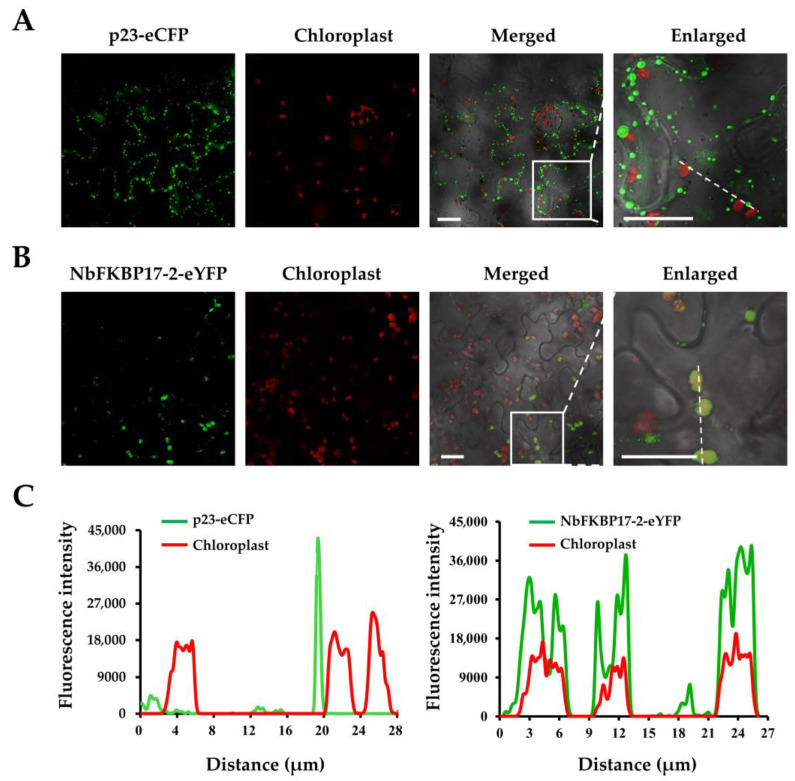
Subcellular localization analyses of citrus tristeza virus (CTV) p23 and NbFKBP17-2 in *Nicotiana benthamiana* cells. (**A**) Localization of CTV p23 and chloroplasts. The red signals represented the autofluorescent signals of chlorophyll in chloroplasts. (**B**) Localization of NbFKBP17-2 and chloroplasts. Confocal images were taken at 48 hpi. Bars, 20 μm. (**C**) The co-localization was further analyzed by overlapping fluorescence spectra, and areas indicated with white dotted lines in enlarged sections of (**A**,**B**) were used for this analysis.

The subcellular localization of p23 in the epidermal cells of *N. benthamiana* leaves was determined using the transient expression vectors pCNC and pCNY. The fluorescence signals in infiltrated leaf cells were visualized under confocal microscopy at 2 dpi. When eCFP was expressed alone, the fluorescence signals visualized for free eCFP located in the cytoplasm and nucleus almost uniformly, as well as in actin filaments close to the nucleus (Figure S3). The fluorescence signal of p23-eCFP accumulated preferentially as parallel spots at plasmodesmata (Figure 2A), similar to what was previously reported [17]. The fluorescent signals of diffused NbFKBP17-2-eYFP appeared as bodies with diameter about 2.5–4.5 μm in chloroplasts, and most of these bodies colocalized with chlorophyll autofluorescent signals (Figure 2B). Further overlapping the fluorescence spectra of p23 with chlorophyll and NbFKBP17-2 with chlorophyll showed that NbFKBP17-2, but not p23, localized to chloroplasts (Figure 2C).

**Figure 3:**
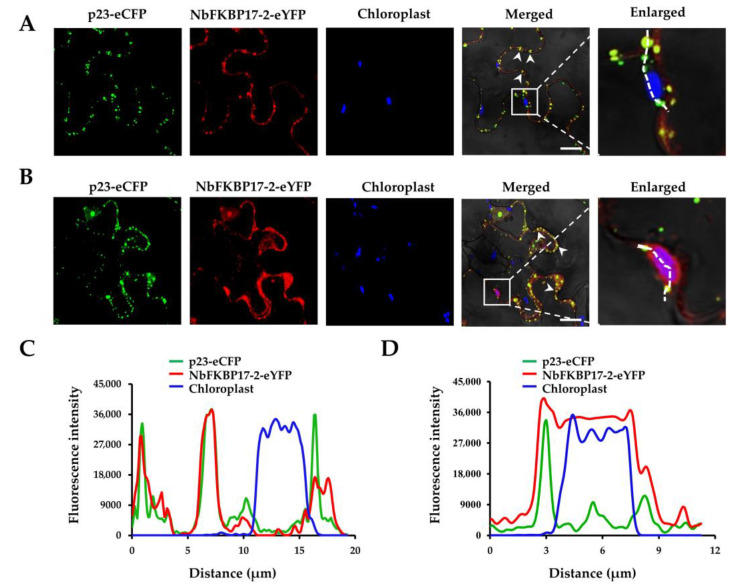
Subcellular co-localization analyses of p23-eCFP and NbFKBP17-2-eYFP in *Nicotiana benthamiana* epidermal cells. (**A**,**B**) represented two different visual fields showing the co-localization of p23 and NbFKBP17-2 at plasmodesmata (PD) (**A**) and partial fluorescent signals of NbFKBP17-2 on chloroplasts (**B**), respectively. The white boxes indicated areas showing a magnified view. Arrows point to PD. The blue signals represented the chlorophyll autofluorescence in chloroplasts. Confocal images were taken at 48 hpi. Bars, 20 μm. (**C**,**D**) showed overlapping fluorescence spectra of eCFP, eYFP and chloroplast signals along the white dots depicted in (**A**,**B**), respectively.

The co-localization features of p23 and NbFKBP17-2 were examined by co-expressing the two proteins in the epidermal cells of *N. benthamiana* leaves. A profound change of NbFKBP17-2-eYFP localization was observed in the presence of p23-eCFP. Most YFP fluorescence signals of NbFKBP17-2-eYFP were translocated from chloroplasts to plasmodesmata and cytoplasm (Figure 3A). The co-localization feature of p23-eCFP and NbFKBP17-2-eYFP was similar to their interaction signals in BiFC assays (Figure 1C). Occasionally, we found the fluorescence signal of p23-eCFP as small dots in a few chloroplasts where NbFKBP17-2-eYFP also presented (Figure 3B). The overlapping fluorescence spectra also showed that p23 translocated NbFKBP17-2 partially from chloroplasts to PD (Figure 3C,D).

### 3.3. NbFKBP17-2 and p23 Were Involved in CTV CP Movement

Since p23 alone and p23 together with NbFKBP17-2 accumulated preferentially at PD as tested above, we suspected that the co-localization features of these proteins might be involved in the virus movement. Considering that many plant viruses move as nucleocapsid, we tested the accumulation type of CTV CP in *N. benthamiana* cells. Results showed that CTV CP alone located at PD, and in nucleus and cytoplasm (Figure S4).

**Figure 4:**
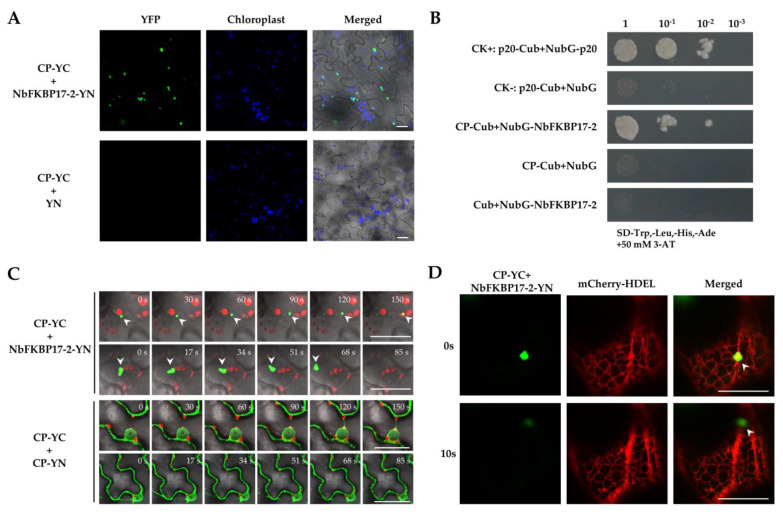
NbFKBP17-2 interacted and co-moved with citrus tristeza virus (CTV) CP in *Nicotiana benthamiana* cells. (**A**) Bimolecular-fluorescence complementation assays. The CP fused to YFP C-terminal (CP-YC) was co-expressed with NbFKBP17-2 fused to YFP N-terminal (NbFKBP17-2-YN) by agroinfiltration on *N. benthamiana* leaves. Blue fluorescence represented chlorophyll autofluorescence. (**B**) Split-ubiquitin yeast two-hybrid assays. CP was fused to the C-terminal half of ubiquitin (Cub), NbFKBP17-2 was fused to the N-terminal half of ubiquitin (NubG). Yeast cell of co-transformed with p20-Cub and NubG-p20 was used as a positive control and p20-Cub and NubG was used as a negative control, respectively. A series of dilutions (1, 10^−1^, 10^−2^ and 10^−3^) were shown at the top of the figure. (**C**) Time-lapse images of CP/NbFKBP17-2 interaction and CP self-interaction. Two panels of each interaction combination were the captured signals in two fields. Red bodies were chloroplasts with chlorophyll autofluorescence signals. (**D**) Co-localization of CP/NbFKBP17-2 interaction complexes with endoplasmic reticulum marker mCherry-HDEL. The arrow heads pointed to CP/NbFKBP17-2 interaction complexes. Confocal images were taken at 48 hpi. Bars, 20 μm.

BiFC assay showed that CP-YC and NbFKBP17-2-YN interacted in *N. benthamiana* leaf cells and YFP fluorescence signals appeared as numerous bodies with variable sizes in the cytoplasm of agroinfiltrated *N. benthamiana* cells at 48 hpi, but did not colocalize with chloroplasts. There was no interaction signal observed for the control combination of CP-YC with unfused YN (Figure 4A). The interaction of CP with NbFKBP17-2 was further investigated by Y2H assay. The yeast cells transformed with paired vectors p20-Cub and NubG-p20 (a positive control), and CP-Cub and NubG-NbFKBP17-2 were able to grow on selective medium (SD-Trp,-Leu,-His,-Ade) complemented with 50 mM 3-AT, while yeast transformants carrying CP-Cub+NubG, and Cub+NubG-NbFKBP17-2 did not grow, indicating that the CP interacted with NbFKBP17-2 (Figure 4B). Surprisingly, time-lapse microscopy observation found that the CP/NbFKBP17-2 bodies with different sizes could rapidly and irregularly move in the cytoplasm (Figure 4C, upper panel), while the self-interaction of CP appeared as uniform distribution in nucleus and plasmodesmata (Figure S5), but did not form bodies in the cytoplasm and chloroplasts (Figure 4C, low panel). Further assays revealed that the interaction complexes moved on the ER (Figure 4D).

**Figure 5:**
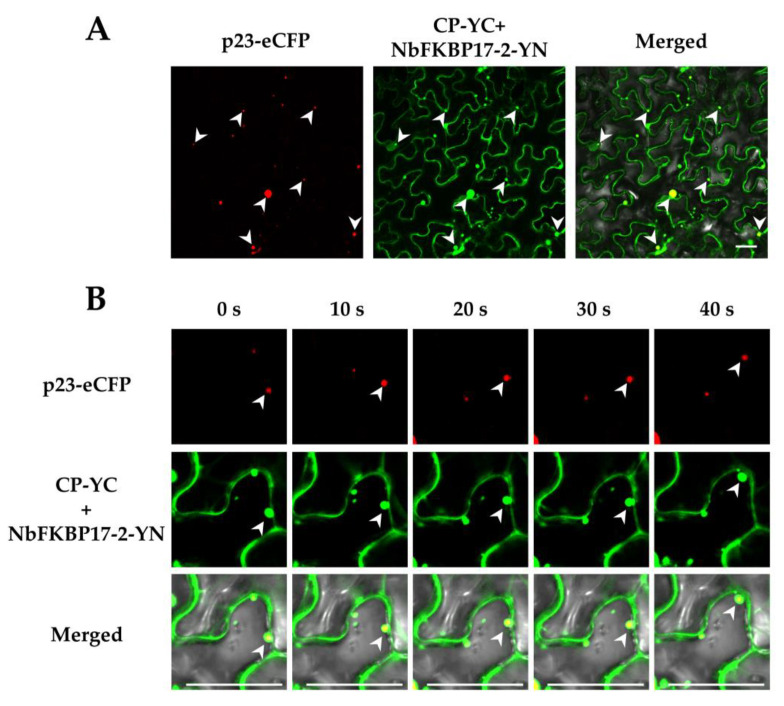
Co-localization (**A**) and integrated time-lapse movement (**B**) of CP/NbFKBP17-2 interaction complexes with p23-eCFP. Arrow heads pointed to p23/CP/NbFKBP17-2 complexes in different channels. Confocal images were taken at 48 hpi. Bars, 20 μm.

Given the fact that NbFKBP17-2 interacts with p23, we suspected that p23 might take part in the movement. The expression vector p23-eCFP and BiFC vectors NbFKBP17-2-YN and CP-YC were co-infiltrated into epidemic cells of *N. benthamiana* leaves. It was found that p23-eCFP and CP/NbFKBP17-2 complexes indeed co-localized as round particles in the cytoplasm and near cell membranes (Figure 5A). Time-lapse observation showed that these particles also moved quickly in the cytoplasm (Figure 5B and Figure S6).

### 3.4. Knock-Down Expression of NbFKBP17-2 in N. benthamiana Led to Decreased CTV Accumulation

**Figure 6:**
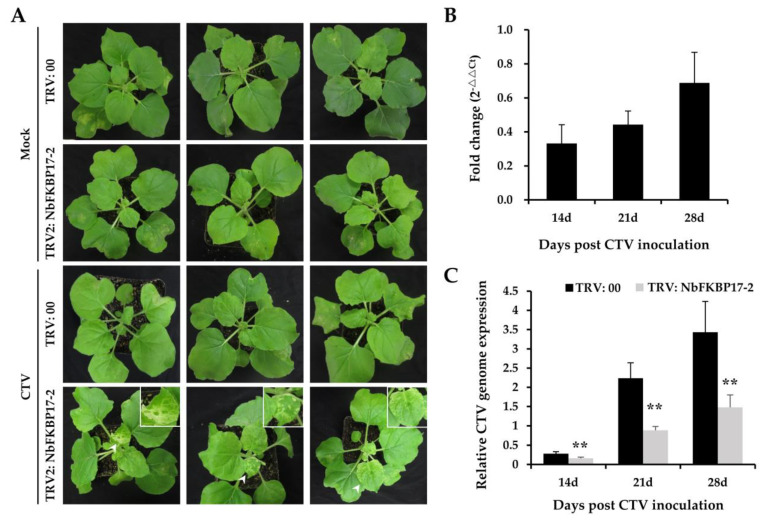
The phenotypes and relative expression levels of NbFKBP17-2 and citrus tristeza virus (CTV) genome in NbFKBP17-2 silenced *Nicotiana benthamiana* plants. (**A**) Phenotypes of TRV: 00 and TRV: NbFKBP17-2 pre-inoculated plants at 14 dpi of mock and CTV. (**B**) RT-qPCR analysis of *NbFKBP17-2* mRNA expression in *N. benthamiana* leaves. The expression levels of *NbFKBP17-2* mRNA were normalized to *NbActin* mRNA levels. (**C**) RT-qPCR analysis of relative expression levels of CTV genome in *N. benthamiana* leaves. Values were means ±SD from three independent experiments. Asterisks denoted significant differences in Student’s *t*-test between the two treatments (two-sided, ** *p* < 0.01).

To examine whether the interaction between p23 and NbFKBP17-2 could influence the accumulation of CTV genome, we constructed a tobacco rattle virus (TRV)-based RNA silencing vector pTRV2-NbFKBP17-2 to knock down *NbFKBP17-2* mRNA via virus induced gene silencing (VIGS). The VIGS vector pTRV2-NbFKBP17-2 was co-infiltrated with plasmid pTRV1 into *N. benthamiana* leaf cells (TRV: NbFKBP17-2). Plants co-infiltrated with the plasmids pTRV2 and pTRV1 were used as negative controls (TRV: 00). At 7 dpi, CTV infectious cDNA clones were infiltrated into *NbFKBP17-2*-silenced and control plants. Mild chlorosis phenotype was observed on Mock-inoculated *NbFKBP17-2*-silenced and control plants at 14 dpi (Figure 6A, panels 1 and 2). At 14 days post CTV inoculation, although the CTV-infected control plants did not show visible disease symptom (Figure 6A, panel 3), the new leaves of CTV-infected *NbFKBP17-2*-silenced plants showed local chlorosis and white blotches (Figure 6A, panel 4). RT-qPCR assays showed that actin mRNA accumulated stably in *N. benthamiana* plants during CTV infection, which was consistent with the results from CTV-infected citrus plants [38,46]. The relative expression level of endogenous *NbFKBP17-2* in *N. benthamiana* was knocked down to about 0.33-, 0.44- and 0.68-fold (Figure 6B), and the accumulation levels of CTV genome in *NbFKBP17-2*-silenced plants were significantly lower than those in control plants (Figure 6C). The results indicated that the decreased expression of endogenous *NbFKBP17-2* reduced CTV accumulation in *N. benthamiana* plants.

## 4. Discussion

CTV p23 protein has multiple functions on the viral replication, movement, suppression of RNA silencing, and pathogenesis [16,17,18,19]. Accordingly, CTV p23 shows specific subcellular localizations in the nucleolus, Cajal bodies, and plasmodesmata. The p23 is a unique protein of CTV. It is suggested that CTV p23 might have evolved for regulating specific interactions of CTV with its citrus hosts [14]. Considering the unique properties of the viral protein, we examined a yeast library of *C. aurantifolia* by using CTV p23 as a bait and identified a CTV p23 interacting protein CaFKBP17-2. Furthermore, we confirmed the interaction between CTV p23 and NbFKBP17-2, a homolog of CaFKBP17-2 from an experimental host *N. benthamiana* for CTV [26]. It was reported that some FKBPs, such as FKBP13 and FKBP16, localized in plant chloroplasts, could regulate photosynthetic membrane assembly [33]. Our results showed that NbFKBP17-2 alone localized in chloroplasts. However, CTV p23/NbFKBP17-2 complexes located at PD as tested in both BiFC and co-localization analyses, suggesting that p23 might change the NbFKBP17-2 subcellular location. CTV p23 is involved in eliciting CTV-like symptoms (vein clearing and chlorotic spots) when it is expressed ectopically as a transgene in several *Citrus* species [18,19]. It is also the CTV determinant of the seedling yellow syndrome in sour orange and grapefruit (*C. paradisi* Macf.) [49]. The ectopic expression of CTV p23 as a transgene does not induce similar symptoms in *N. benthamiana* [19], but expression CTV p23 from a PVX sgRNA is able to incite symptoms in *N. benthamiana* [50]. Thus, the interaction between p23 and NbFKBP17-2 resulting in the changed subcellular localization of NbFKBP17-2 might affect the leaf cell photosynthesis, then induce disease like symptoms [14].

The intracellular and intercellular movement of a plant virus is necessary for the virus systemic infection [51,52]. Except for movement proteins to be responsible for viral movement [53,54,55], other proteins are also known to be involved in the intracellular movement of some viruses. The proteins of some viruses, such as the 126-kDa replication protein of tobacco mosaic virus [56], the membrane-associated P3 of tobacco etch virus, P3N-PIPO of turnip mosaic virus [57,58], the P6 of CaMV [59], and the N protein of tomato spotted wilt tospovirus [60], can form mobile inclusion bodies involved in the virus intracellular movement. The viral coat proteins have multiple functions and are involved in almost every stage of the viral infection cycle [20]. The interaction of host CHUP1 with CaMV P6 can help the viral inclusion bodies moving inside plant cells along microfilament to reach to plasmodesmata [60]. In this study, we found that the interaction complex CP/NbFKBP17-2 could rapidly move in the cytoplasm of agroinfiltrated *N. benthamiana* cells without CTV infection. Since CTV-CP and NbFKBP17-2 alone located in cytoplasm and chloroplasts, respectively, the interaction might occur in cytoplasm before NbFKBP17-2 transportation into chloroplasts and be necessary for CP transportation in cytoplasm of plant cells. In addition, although we found the moveable CP/NbFKBP17-2 bodies on the ER membrane, the exact trace of the bodies was difficult to be captured since they moved at different layers of the cell. The association of ER structures with the movement action needs an extensive study. The movement proteins encoded by some plant RNA viruses may bind to the viral replication complex and facilitate their association with plasmodesmata and the subsequent viral spread [61]. CPs from a number of plant RNA viruses are required for effective cell-to-cell and long distance movement [54,62]. Viruses in the genera *Cucumovirus*, *Potyvirus*, *Potexvirus*, *Comovirus,* and some closteroviruses require their MPs and CPs for both cell-to-cell and systemic movement of the viral genomes [62,63,64]. CTV p23 accumulates preferentially at plasmodesmata and facilitates CTV cell-to-cell movement [65]. Our results showed that the p23 could translocate the CP/NbFKBP17-2 complexes from cytoplasm to cell membranes, which provide a new insight into a host protein involved in intracellular trafficking of the CP and the unique protein p23 of CTV. The p33 is another unique non-conserved and PD localized protein of CTV [61], recent study demonstrated interactions of p33 with three proteins CP, p20, and p23, and the four proteins colocalized in the CTV replication factories. In addition, p33 and p23 colocalized as a number of spots on plasma membrane, as well as in a few vesicle-like structures. Then, the p23/NbFKBP17-2/CP motile complexes may function on facilitating the movement of a ribonucleoprotein replication complex to the PD. Further understanding the roles of p33 in the movement of the p23/NbFKBP17-2/CP complexes will help reveal the CTV movement mechanism.

The knocked-down expression of NbFKBP17-2 in *N. benthamiana* does not result in a visible phenotype. However, the decreased expression of NbFKBP17-2 could reduce CTV titer in *N. benthamiana*. In some *N. benthamiana* plants with decreased NbFKBP17-2 expression, CTV infection causes severe chlorosis of new leaves, suggesting that the hijack of NbFKBP17-2 by CTV p23 could greatly decreased NbFKBP17-2 accumulation in chloroplasts, which might result in the reduction of chlorophyll. Thus, like a recently reported p23-interacting host protein GAPDH [30], the interaction of NbFKBP17-2 with CTV p23 might facilitate CTV infectious cycle.

Collectively, our findings provide the first evidence that the p23 of CTV has the ability of intracellular trafficking with the viral coat protein by interaction with a host protein.

## Data Availability

The data presented in this study are openly available in article and Supplementary Materials.

## Supplementary Materials

The following are available online at https://www.mdpi.com/article/10.3390/cells10040934/s1, Figure S1: Time-lapse images of p23/NbFKBP17-2 interaction; Figure S2: Bimolecular fluorescence complementation assay on the interactions between p23-YC and unfused N-terminal fragment of YFP and between FKBP17-2-YN and unfused C-terminal fragment of YFP in *Nicotiana benthamiana* cells; Figure S3: Subcellular localization analyses of enhanced cyan fluorescent protein (eCFP) in *Nicotiana benthamiana* cells; Figure S4: Subcellular localization analyses of CTV CP in *Nicotiana benthamiana* cells; Figure S5: Bimolecular-fluorescence complementation assay of CTV CP self-interaction in *Nicotiana benthamiana* cells; Figure S6: Dynamic image of colocalization of CP/NbFKBP17-2 interaction complexes with p23-eCFP, Table S1: Primers used in this study.
